# 

**Published:** 1818

**Authors:** 


### College Certificates.

*Veterinary College*, 12*th July*, 1815.

THESE are to certify, that Mr. J. Carver has attended the Veterinary College as a resident pupil for three years, and having been examined by us, we consider him as qualified to practise the Veterinary Art.

HENRY CLINE, Surgeon,
WILLIAM BABINGTON, M.D.,
ASHLEY COOPER,
J. COOK, M.D.,
G. PEARSON,
HENRY CLUI, Jun.,
EDWARD COLEMAN, Professor,
WM. SEWELL, Assistant Professor and Treasurer.

*Theatre of Anatomy, Physiology, Pathology, and Surgery, October*. 1815.

By Mr. Wilson, Mr. Charles Bell, and Mr. Brody.

This to certify, that Mr. J. Carver, Veterinary pupil under me, has attended a course of lectures on the human subject, and chymistry, under sir H. Davy, Jun.

EDWARD COLEMAN, President,
WM. SEWELL, Assistant Professor.

*Royal Veterinary Medical Society, July* 12,1815.

We hereby certify, that Mr. J. Carver is a member of the London Veterinary Medical Society, and that his observations have contributed to the advancement of Veterinary knowledge.

Signed by order of the Society.

EDWARD COLEMAN,
WM. SEWELL, V. P.

S. DUFFIELD, Secretary.
*To Mr. J. Carver, V. S.*

### Agricultural Society.

The above Veterinary diplomas have been examined and approved by the President, Vice President, Secretary, and Members of the Philadelphia Agricultural Society. R. VAUX, Secretary.

Understanding that several malicious and ungrounded reports having been in circulation, respecting Dr. Carver, Veterinary Surgeon, having killed a mare of mine, which he never attended, I deem it my duty, as a fellow citizen and patron of the Veterinary science, to contradict any such evil report as may have a tendency to injure the professional talents of a man, whose services I not only estimate, but shall avail myself of, as occasion may require.

JOHN TOMLINSON.

Philadelphia, 5th mo. 16th, 1817

---

Having attended, at the request of the parties, an investigation of the circumstances which led to the death of Mr. Chancellor's horses, we do certify that nothing came to our knowledge which ought to affect any estimate which might previously have been entertained of the skill and abilities of Mr. Carver as a practitioner of Veterinary Medicine.

N. CHAPMAN, Professor of Anatomy and Mat. Med.
THOS. T. HEWSON, Prof. of Comparative Anatomy.

---

*** The Students of the University of Pennsylvania are respectfully invited, in the ensuing winter, to attend a Course of Lectures on the Foot of the Living Horse; and the various diseases attendant on Quadrupeds in general.

### HEADS OF SUBJECTS

TO BE TREATED OF IN

### DR. CARVER'S PROPOSED PUBLICATIONS.

---

*,* In order that the public may be put in possession of all the necessary information in Veterinary Science, Dr. C. will embrace the earliest opportunity for treating on the various subjects here detailed; selecting for his first publications those which may, at the present time, be considered of the greatest importance to this rising country.

---

### OF THE HORSE.

General management of the Horse, including observations on the Stable; its situation, size, cleanliness, ventilation, temperature, &c.

### OF STABLE DUTY.

Arrangement of Stable Duty; bad consequences of its neglect; proper time of doing it, &c.

### OF FOOD.

Proper mode of Feeding with different articles, including soft food, farinaceous grains, oats, Indian corn, &c. Consequences of hard food; showing the great advantages that would be derived from the more general introduction of soft food, such as steamed potatoes, carrots, Swedish and Russian turnips, bran, shorts, and particularly bruised corn of every description. On the impropriety of stuffing horses with so much dry hay; with observations on a more politic mode of cutting it for mixing with other food.

### ON LABOUR.

Remarks and observations on Labour; and the proper mode of appropriating it among other draught horses and oxen; time in the yoke; choice of cartmen and drivers; with consequences of improper treatment after labour.

### ON DISEASES.

Injurious customs of bleeding and physicking horses at particular seasons of the year; including an essay on the use and abuse of purgatives. Prevention of disease, reduced to the heads of Air, Dressing, Feeding, Watering, and Light; with observations on the necessity of Ventilation.

*On Diseases of the Liver.*—Jaundice.

*On Diseases of the Kidneys.*—Suppression of the Urine.

*Diabetes, and Diseases of the Bladder.*—Structure and economy of the Bladder—Inflammation of the Bladder; causes, symptoms, best mode of treatment, and cure; operation, how performed, to draw the water from the Chest, Bladder, &c. Difference of structure in the horse and man; difference of operating. Of Calculi.

*Diseases of the Intestines.*—Of the Cholic, or Gripes—Diarrhœa, or Looseness. Sleepy and Mad Staggers—Stomach Staggers; its causes, symptoms, and cure.

*Of Glanders.*—Glanders in the human subject; also in Swine. Comparison between Farcy, Glanders, and the Venereal disease.

*Inflammation of the Stomach.*—Inflammation of the Lungs—Description of the necessary operations, and how performed in this fatal disease, made simple and easy.

*Diseases of the Eye.*—Of the Eye, its structure, *&c.* Of Vision, how performed. Of Opthalmy, Cataract, or Moon Blindness, as so called by farriers. Of Gutta Serena, or Glass Eyes, *&c.*

### OF THE PULSE.

Mode of feeling and judging of the Pulse in different diseases. Of the circulation in general. How to know when to bleed and when not, by the action of the pulse. How to judge of the blood when first drawn, and when coagulated by the action of oxygen or the external air.

### ON THE ORGANS OF RESPIRATION.

Of the Trachea, or Windpipe; its structure—Roaring—Broken Wind—Strangles—Catarrh, or Cold—Chronic Cough; its treatment and cure.

### OF THE EPIDEMIC DISEASE,

Which has been so prevalent for these two years past in this city, the suburbs, and on the Lancaster line.

### ON SWEATING.

A cooling and salutary process in the above and other diseases. How and with what medicines this secretion is to be drawn to the surface of the body, as also by means of Dr. Carver's Patent Medical Vapour Bath.

### ON INFLAMMATION, AND TREATMENT OF WOUNDS IN GENERAL.

On Inflamed Veins, and danger from bleeding; how to be treated—The Suppurative process in wounds—Punctured wounds—Wounds of the Joints; great danger attendant on; only one mode of cure, and that explained—Contused and lacerated wounds—Ulcers, Fistula, and Pole Evil; treatment and cure pointed out—Hæmorrhage, or bleeding in general—Sprains.

### ON THE FOOT IN GENERAL.

The horny Hoof; its structure and use—The horny and sensible Laminæ; their elastic folds; 500 of each surrounding the horny box; their structure and arrangement—Whilst under inflammation, its treatment and cure—The Coffin Bone—Horny and sensible Sole, their uses—The Frog; its use as an elastic stop to expand the heels explained—Corns, Contraction, Thrush, Canker, Sand-crack, Quittor, &c. &c.; their symptoms, treatment and cure, on a new principle never before introduced in this country, by the author's Patent Veterinary Medical Bath.

### ON DISEASES OF THE LEGS.

Best mode of treatment and cure, together with the necessary operations, and how performed.

### ON HOBBLES.

Description of the Patent Hobbles, improved by the author, for casting horses without injury, for the performance of the necessary operations.

### ON CASTRATION.

College mode of performing it on new principles, and also of operating for Curbs, Bony and Blood Spavins, Thoroughpin, Wind-galls, &c. &c. by actual cautery—together with observations on general and local bleeding.

### OF HORSE BALLS, DRINKS, AND CLYSTERS.

How administered, pointing out the danger of giving drink and drenches to horses—not the same effect on horned cattle.

### ON OPERATIONS.

Particular directions for performing the most important operations in farriery, such as Firing, Blistering, Rowelling, Bleeding, &c. agreeably to the College mode of practice—(with a plate.)

### ON DRAUGHT HORSES.

Best management of draught horses, particularly those used in drays and carts in and about the city of Philadelphia.

---

### ON THE DISEASES

OF

### *COWS, SHEEP, SWINE, AND DOGS;*

With additional Observations on their Diseases, made during my residence at the Veterinary College.

---

### On the Diseases of Cows.

Chapter I.—Inflammatory Fever, or general Inflammation; by the common farrier known by the various names of Quarter Ill, Quarter Evil, Joint Felon, and many others equally absurd and unmeaning.

Chap. II.—Fever, Putrid, Malignant, Epidemic, Murrain, or Pest.

Chap. III.—Catarrh, or Cold, Catarrh Epidemic, Influenza, Distemper, or according to Dr. Clayton, Felon.

Chap. IV.—Inflammation of the Lungs.

Chap. V.—Inflammation of the Stomach.

Chap. VI.—Inflammation of the Bowels.

Chap. VII.—Inflammation of the Liver.

Chap. VIII.—Inflammation of the Kidneys.

Chap. IX.—Inflammation of the Bladder.

Chap. X.—Inflammation of the Womb.

Chap. XI.—Inflammation of the Brain.

Chap. XII.—Fog Sickness, Hoven, or Blown.

Chap. XIII.—Gripes, or Flatulent Cholic.

Chap. XIV.—Indigestion, or Loss of the Cud.

Chap. XV.—Jaundice, or Yellows.

Chap. XVI.—Diarrhœa, Looseness, Scouring, and Rot.

Chap. XVII.—Red Water, or Bloody Urine.

Chap. XVIII.—Dysentery, or Bloody Ray.

Chap. XIX.—On the Management of Cows when near their time of Calving.

Chap. XX.—Inflammation and Swelling of the Udder.

Chap. XXI.—On Wounds, Strains, and Bruises.

Chap. XXII.—On the Parturition of the Cow and Sheep.

### On the Diseases of Sheep.

Chapter I.—The Rot, the Foot Rot, the Scab, Red Water, Dysentery, or Braxy, Diarrhœa, or Scouring.

Chap. II.—Inflammatory Fever, General Inflammation, Blood, or Blood Striking.

Chap. III.—Hydrocephalus, Hurdy, Goggles, Staggers, Turnsick, &c.

---

### On the Diseases of Dogs and Swine.

Chapter I.—Distemper, Worms, Madness, or Hydrophobia, Mange Canker in the Ears, &c.

Chap. II.—Observations on the Diseases of Swine.

To conclude with remarks and observations on the parturition of the Mare, the Cow, and Sheep, with the attention and medicine necessary for their relief in preternatural presentation.

